# Advances in Development of Radiometal Labeled Amino Acid-Based Compounds for Cancer Imaging and Diagnostics

**DOI:** 10.3390/ph14020167

**Published:** 2021-02-21

**Authors:** Mária Bodnár Mikulová, Peter Mikuš

**Affiliations:** 1 Department of Pharmaceutical Analysis and Nuclear Pharmacy, Faculty of Pharmacy, Comenius University in Bratislava, Odbojarov 10, 832 32 Bratislava, Slovakia; mikulova43@uniba.sk; 2 Toxicological and Antidoping Center (TAC), Faculty of Pharmacy, Comenius University in Bratislava, Odbojarov 10, 832 32 Bratislava, Slovakia

**Keywords:** amino acid, peptide, bifunctional chelating agent (BFCA), radiolabeling, cancer, receptor, imaging

## Abstract

Radiolabeled biomolecules targeted at tumor-specific enzymes, receptors, and transporters in cancer cells represent an intensively investigated and promising class of molecular tools for the cancer diagnosis and therapy. High specificity of such biomolecules is a prerequisite for the treatment with a lower burden to normal cells and for the effective and targeted imaging and diagnosis. Undoubtedly, early detection is a key factor in efficient dealing with many severe tumor types. This review provides an overview and critical evaluation of novel approaches in the designing of target-specific probes labeled with metal radionuclides for the diagnosis of most common death-causing cancers, published mainly within the last three years. Advances are discussed such traditional peptide radiolabeling approaches, and click and nanoparticle chemistry. The progress of radiolabeled peptide based ligands as potential radiopharmaceuticals is illustrated via novel structure and application studies, showing how the molecular modifications reflect their binding selectivity to significant onco-receptors, toxicity, and, by that, practical utilization. The most impressive outputs in categories of newly developed structures, as well as imaging and diagnosis approaches, and the most intensively studied oncological diseases in this context, are emphasized in order to show future perspectives of radiometal labeled amino acid-based compounds in nuclear medicine.

## 1. Introduction

Over past 20 years, in the field of nuclear medicine, substantial progress has been made in the development of novel radiopharmaceuticals and radiolabeled agents for diagnosis and therapy of various diseases. Nowadays, a great emphasis is put on a synthesis and study of radiolabeled amino acid-derived biomolecules with a selective distribution and binding to target structures in living cells and tissues, i.e., enzymes, transporters, or peptide receptors. This allows targeted therapy and diagnostic evaluation of pathological changes in many fields, such as oncology, neurology, endocrinology, cardiology, and also investigation of inflammation processes or infection.

Especially, malignant tumor diseases are of the biggest interest because of their increasing global incidence, and placing second in the causes of death. The effect of target-specific radiolabeled compounds is often mediated through binding with high affinity to specific protein structures (e.g., active places in enzymes or receptors). Many of these structures are overexpressed in diseased cells compared to their absence or lower density under physiological conditions. Since that, such radiolabeled compounds represent effective probes in a recognizing and visualizing tumor cells in their early stage. All types of malignant solid tumors often exhibit lower oxygenation levels than their original tissues resulting in a hypoxic state, which impacts on the reduced effectiveness of tumor therapy and propagation of metastasis. Hence, there is an urgent need to enhance detection approaches for monitoring various tumor types, including hypoxic cancer lesions. In this field, amino acid-based target-specific radiopharmaceuticals have become significant tools in modern oncology allowing cancer imaging on molecular and cellular level [1].

In order to utilize biomolecules for imaging and diagnosis, they must be properly labeled. Metal radionuclides belong to the most powerful and the most employed labels in nuclear medicine. In the group of metallic radioisotopes, gamma and positron emitters, namely copper-62, copper-64, gallium-67, gallium-68, indium-111, and technetium-99m have proved to be the most suitable for nuclear research and clinical application [2,3]. Apparently, other potential radionuclides such as zirconium-89, yttrium-86, and cobalt-55 have been included in recent studies since these have become more readily available with high purity. A diversity of synthesis strategies, radiolabeling approaches, modified chelators, and linkers has been investigated and developed to reach the optimized target-specific radiolabeled compounds, with proper characteristics for cancer imaging and therapy. All of these crucial components of radiolabeled compounds are the subject of many review papers, with a focus on the chemistry of metallic radionuclides [4,5,6], chelators, and linkers [7,8,9,10,11,12], as well as onco-specific peptidic biomolecules [2,13,14,15,16].

The aim of this review is to summarize and critically evaluate state-of-the-art approaches and the most significant outputs related to the development of target-specific radiometal labeled biomolecules for imaging of severe tumor types and tumors with an increased incidence. Recent advances in synthetic approaches and radiometal-labeling strategies of amino acid-based biologically active molecules, including most employed peptide families and receptors such as somatostatin, cholecystokinin/gastrin, bombesin, integrins, and hypoxia endogenous markers, as well as inhibitors of prostate-specific membrane antigen and fibroblast activation protein, are highlighted in order to demonstrate perspectives in cancer diagnostics with amino acid-based radiopharmaceuticals.

## 2. Basic Characteristics of Conventional Metal Radionuclides and Chelators Currently Used in Nuclear Medicine

**Figure 1:**
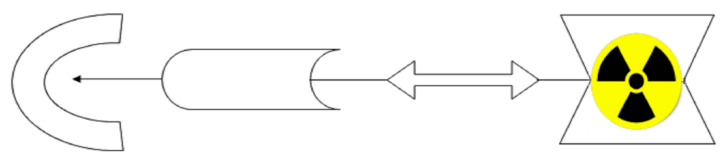
Basic scheme of a potential target-specific radiopharmaceutical.

Radiometallic compounds with targeted biodistribution and binding in the human body (i.e., target-specific) include in their structure: (i) biomolecules as a crucial biodistribution component (specific to receptor); (ii) a linker as a connecting component preserving specificity of biomolecule when attaching; (iii) a bifunctional chelating agent (BFCA); and (iv) metal radionuclides (see Figure 1). Basic characteristics of the most important or most frequently used representatives in the group of conventional metal radionuclides and BFCA are briefly discussed in Section 2.1 and Section 2.2, respectively. Discussion is led in general point of view or, if appropriate, with respect to amino acid based biomolecules.

### 2.1. Metal Radionuclides

In general, a diagnostic radioprobe contains a gamma emitting radionuclide for SPECT imaging or a positron emitting radionuclide for PET imaging. Basic parameters of the most common metallic radionuclides for diagnostic nuclear medicine are summarized in Table 1.

Nuclear medicine research is currently focused on development of a highly potent target-specific biomolecule labeled with positron emitters (predominantly gallium-68, but also zirconium-89, copper-64, and others). Anyway, there is still a leading position of technetium-99m in diagnostic clinical practice. In research, a prognosis for the development of Tc-radiopharmaceuticals is also quite positive due to novel modifications of BFCA and linkers continuously presented and developed for SPECT imaging.

### 2.2. Bifunctional Chelating Agents (BFCA)

Since the metallic radionuclides themselves cannot be utilized in a direct radiolabeling of amino acid-based target-specific compounds (peptides, proteins), it is necessary to develop bifunctional chelating agents (BFCA) [12]. An appropriate BFCA can properly attach both a metallic radionuclide and a biomolecule as well. The double function of BFCA helps the biomolecule to retain its receptor specificity and, thus, to match metal properties with the intended utilization in the imaging/therapy of various diseases. The choice of BFCA takes into account the oxidation state and nature of the metallic radionuclide. The optimal BFCA should provide thermodynamically stable and kinetically inert complexes, rapid reaction (at low temperatures and concentration), flexible conjugation chemistry, and should be easily accessible [17,18].

Various acyclic and cyclic BFCA have been introduced into (potential) radiopharmaceuticals. Traditional examples of acyclic and cyclic BFCA are discussed in Section 2.2.1 and Section 2.2.2, respectively, while the most commonly used BFCA in radiolabeling with a particular diagnostic radiometal including newer developed chelators in Section 3.2.1, Section 3.2.2, Section 3.2.3, Section 3.2.4 and Section 3.2.5.

#### 2.2.1. Acyclic BFCA

**Figure 2:**
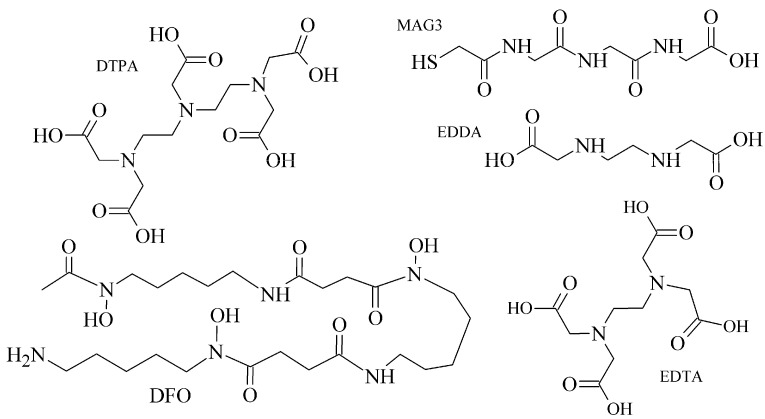
Chemical structures of the most common acyclic chelators as a base of BFCA.

The polyaminopolycarboxylic acids-derived BFCA, such as DTPA, EDDA, EDTA, as well as tripeptide MAG3 (Figure 2), are the most commonly used acyclic BFCA containing hard donor atoms (N, O) in their molecule to form the coordination bond with metallic radionuclide. Another acyclic chelator, a siderophore-based desferrioxamine-B (DFO) has been utilized for effective radiolabeling of biomolecules with a metal. The thermodynamic stability and inert kinetics of a formed complex is unique and influenced by properties of both, a metal radionuclide as well as a BFCA. A significant advantage of the acyclic BFCA is faster metal binding kinetics, resulting in a faster radiolabeling procedure [17]. On the contrary, acyclic BFCA form less stable complexes than cyclic ones due to a higher interaction probability and more fixed geometry of donor atoms in the cyclic BFCA [18].

#### 2.2.2. Cyclic BFCA

**Figure 3:**
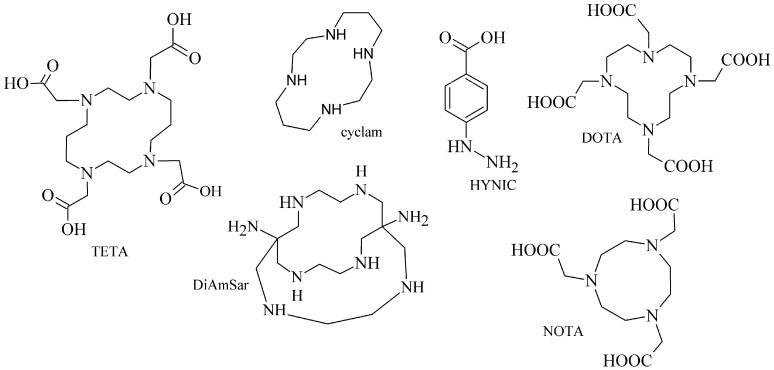
Chemical structures of the most common cyclic chelators as a base of BFCA.

The cyclic BFCA containing macrocycle such as DOTA, NOTA, TETA, and their derivatives as well as various structurally related analogues (for selected representatives see Figure 3) are holding an important position in syntheses of radiolabeled peptide-based compounds over a long period. Several new next generation cyclic chelators or chelators derived from traditional ones with improved properties have been developed over past decade such as PCTA, AAZTA, TRAP, THP, and fusarinine C [19]. As mentioned above, cyclic BFCA are beneficial generally by providing more kinetically inert and thermodynamically stable complexes with metal radionuclides. In order to obtain complexes with enhanced stability, several properties have to be considered such as hard and soft acid and base concept, a higher number of donor atoms providing a better steric fixation of complex, and a proper cavity size for the encapsulation of the whole size of metal ion in a tight structural arrangement.

DOTA is considered as the golden standard of chelators owing to its high kinetic stability. Several types of DOTA-derived chelators have been developed to bind with target peptide biomolecules, i.e., protected DOTA forms, active DOTA esters, and DOTA- derivatives with a coupling moiety [20]. Concerning NOTA, derivatives with aminocarboxylic acids have been applied as BFCA, e.g., NODAGA (with glutaric acid), NODASA (with succinic acid), or NODAPA (with *p*-phenylacetic acid) [21]. Abrams and co-workers used 6-hydrazinopyridin-3-carboxylic acid, in short HYNIC, for radiolabeling of a polyclonal antibody with technetium-99m [22]. Ever since, HYNIC has become the most convenient chelator for ^99m^Tc-labeled peptides and antibodies. Other chelators related to bisthiosemicarbazone [23,24], cyclam [25,26], and sarcophagine [27,28] have been increasingly studied to improve kinetic inertness and stability of complexes, especially those with copper isotopes.

## 3. Complexes and Radiolabeling Approaches for Target-Specific Peptide Molecules

The amino acids, main peptide and protein building blocks, play an important role essentially in all biological processes. Radiolabeled amino acids (AA) have become actively studied, owing to the role of their transporters in the tumor environment. Studies indicated that AA transporters, which recognize, bind and carry amino acids across the plasma membrane, serve not only to maintain nutritional requirements, but also to accumulate particular amino acids in specific cells [29,30].

Analogically, radiolabeled peptides as amino acid-based biomolecules are in the center of interest in the field of nuclear medicine and pharmacy because their biological action is mediated upon selective binding to specific peptide receptors and transporters overexpressed in numerous tumor cells. These receptors have shown potential as a molecular target for tumor imaging or targeted therapy with radiolabeled peptides (for the most important onco-specific peptide receptors and radiolabeled peptides see Section 4). The following Section 3.2, Section 3.3 and Section 3.4 are dealing with current radiolabeling approaches used for peptides and showing corresponding complex structures.

### 3.1. Peptides as Target-Specific Molecules and Their Synthesis

**Figure 4:**
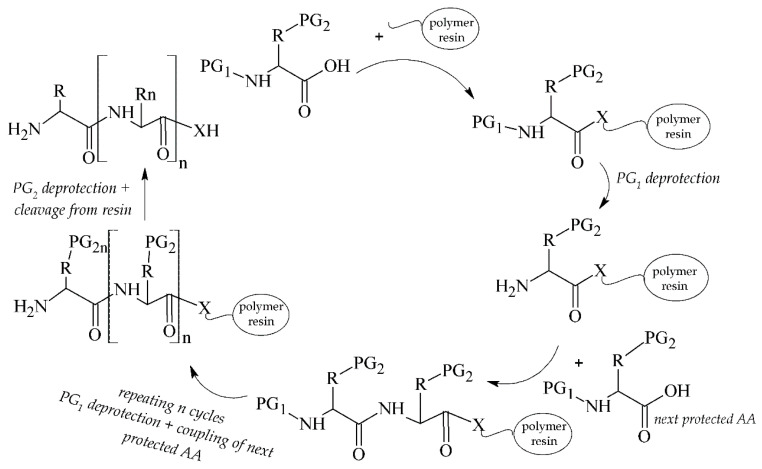
Scheme of solid phase peptide synthesis (SPPS). PG_1_ = temporary protecting group; PG_2_ = semi-permanent protecting group; X = N/O.

Peptides can be simply synthesized by a solid phase peptide synthesis (SPPS) [31,32] and modified to obtain optimized pharmacokinetic properties. The synthetic procedure can be carried out manually [33], e.g., in syringes, or automatically in commercial synthesizers [34]. A general pattern for the solid-phase peptide synthesis is depicted in Figure 4.

The advantages of peptides over proteins and antibodies can be seen in a preparation method, a rapid blood clearance, and the ability to tolerate harsh reaction conditions. On the other hand, a rapid enzymatic degradation by physiological peptidases is a significant limitation of peptides. Anyway, there are several strategies how to avoid this drawback including structural modifications of the C-/N-terminus, incorporation of a PEG linker or D-/unnatural AA, and cyclization [35].

### 3.2. Conventional Radiolabeling Approaches of Peptides with Metallic Radionuclide

The choice of a radiolabeling approach depends on radionuclide nature and a bioactive molecule. A direct labeling strategy is more difficult to be used for a metal attachment to biomolecules (e.g., peptides, proteins). Since the direct approach provides low site-specific and unstable products, and is applicable only to antibodies and their fragments, an indirect labeling method with BFCA has become preferred for a metal-peptide linkage. The usage of BFCA often requires multistep synthesis and involves non-specific interactions, thus a searching for new strategies with more effective incorporation of BFCA into peptide biomolecules has led to innovative approaches in the radiochemistry field such as click reactions (Section 3.3) and radiolabeled nanoparticles (Section 3.4). Modified BFCA and linkers may improve pharmacokinetic properties of a radiolabeled compound. Conventional radiolabeling approaches and chemical structures of corresponding complexes with the most frequently used metal diagnostic radionuclides are discussed in following Section 3.2.1, Section 3.2.2, Section 3.2.3, Section 3.2.4 and Section 3.2.5.

#### 3.2.1. Radiolabeling of Peptide-Based Compounds with Technetium-99m

**Figure 5:**
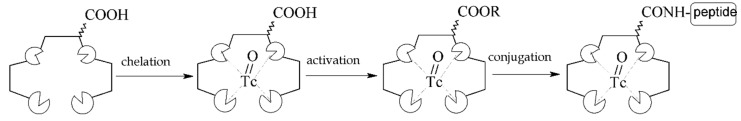
Scheme of the pre-labeling procedure with technetium-99m (adapted according to [3]).

Technetium-99m has been the most frequently used radionuclide in nuclear medicine since the ^99^Mo/^99m^Tc generator development in 1957. Indirect labeling approaches, such as pre-labeling (labeling before conjugation with biomolecule) or post-labeling (labeling after conjugation with biomolecule), are of the routine for ^99m^Tc-coordination. The pre-labeling procedure (Figure 5) is very useful in research to prove the concept and define the chemistry, contrary to a clinical use because of a long lasting radiosynthesis and hardly accomplished kit formulation [3].

**Figure 6:**
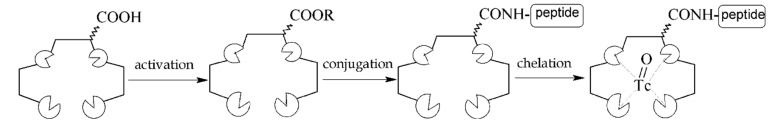
Scheme of the post-labeling procedure with technetium-99m (adapted according to [3]).

The post-labeling procedure (Figure 6) is the most widely used for a synthesis of target-specific peptide radiopharmaceuticals. 

Technetium chemistry, its cores and complexes, have been thoroughly reviewed in recent years [4,6,36,37]. The most frequently studied BFCA for Tc-complexes are summarized in Table 2. In past few years, [^99m^Tc]Tc-HYNIC has been the most commonly used core for the conventional radiolabeling of bioactive peptides for tumor imaging such as RGD peptides [38,39], α-MSH peptide analogues [40,41], bombesin analogues [42,43], substance P analogues [44], or glucagon-like peptide analogues [45].

#### 3.2.2. Radiolabeling of Peptide-Based Compounds with Gallium-68

Gallium is represented by the oxidation state III+ in aqueous solution and acts as a hard Lewis acid. It binds to hard Lewis bases such as nitrogen and oxygen donor groups of carboxylates, hydroxamates, amines [17]. It can be relatively easy hydrolyzed at pH 4–7 [49]. Gallium forms complexes with the maximum coordination number of 6 in a pseudo octahedral geometry, but four- or five-coordinate complexes are also formed [17,49] For a ^68^Ga-labeling procedure, well-known representatives and the most frequently used BFCA are derived from 1,4,7-triazacyclononane and 1,4,7,10-tetraazacyclododecane, e.g., DOTA and NOTA, including their recently developed derivatives such as TRAP, PCTA, NOTP, and THP and DATA, among others (see examples in Table 3).

The ^68^Ga-labeled biomolecules have been studied for somatostatin receptor-positive tumor imaging over a long period [58,59,60] with several highly potent agents in clinical trials or one already approved. Current studies with gallium-68 have followed up various malignancies with prostate-specific membrane antigen (PSMA) and fibroblast activation protein (FAP) [55,61,62].

#### 3.2.3. Radiolabeling of Peptide-Based Compounds with Indium-111

Indium-111 has several properties for coordination chemistry with gallium-68 in common. The only stable oxidation state of indium-111 is III+ and acts as the Lewis acid, but softer donor groups can be offered to create seven or eight-coordinated complexes [49]. The ionic radius of indium-111 (0.92 Å) is significantly larger than that of gallium-68 (0.65 Å) what results in different coordination in macrocycles. The DTPA- and DOTA-based chelators usually in *t*-butyl forms are generally the most employed for the ^111^In-labeling (see Table 4) [63].

Studies covering ^111^In-labeled biomolecules are aimed at somatostatin receptor imaging [66], glucagon-like peptide receptor [67,68], gastrin-releasing peptide receptor [69], or integrins [70].

#### 3.2.4. Radiolabeling of Peptide-Based Compounds with Copper-64

The most stable oxidation state of copper in aqueous solution is II+ creating complexes with donor atoms such as amine-, imine- and pyridine-N, carboxylate-O, and thiol-S [17]. Although the copper chelation chemistry has been thoroughly reviewed [13,18,49,71], there is still a challenge in the development of in vivo stable Cu-BFCA complexes due to labile character of Cu(II). The design of copper radiopharmaceuticals has put emphasis on polyaza-macrocycles derived BFCA (see Table 5). Due to only moderate stability of [^64^Cu]Cu-DOTA-labeled biomolecules under in vivo conditions and high liver accumulation, a number of cross-bridged cyclam derivatives were developed to form more stable ^64^Cu-complexes [25,26,72].

^64^Cu-labeled compounds have been included, mostly in the studies of tumors with overexpressed gastrin-releasing peptide [73,74] and α_ν_β_3_ integrin receptors [75,76], and prostate-specific membrane antigen [77].

#### 3.2.5. Radiolabeling of Peptide-Based Compounds with Zirconium-89

Zirconium is a metal belonging to the group IV that exists primarily in +IV oxidation state in aqueous media. This cation is relatively large, acts as the hard Lewis acid and prefers anionic oxygen donor groups to create complexes with high coordination number [86]. Depending on pH, oxides and hydroxides of zirconium form polynuclear species upon hydrolysis at very low pH and mononuclear hydrolysis species at pH between 0 and 2 [87].

In order to effectively utilize zirconium-89, various chelators have been employed such as DOTA, DTPA, as well as the most successful desferrioxamine B and 3-hydroxypyridin-2-one (2,3-HOPO) derivatives (see Table 6).

Zirconium-89 has been applied mostly in labeling of monoclonal antibodies for PET imaging of immune-based strategies [88], but there has been a progress in the design of ^89^Zr-labeled small peptide PSMA-inhibitors for prostate cancer imaging lately [89].

### 3.3. Radiolabeling Approaches of Peptides with Metallic Radionuclide Based on Click-Chemistry

**Figure 7:**
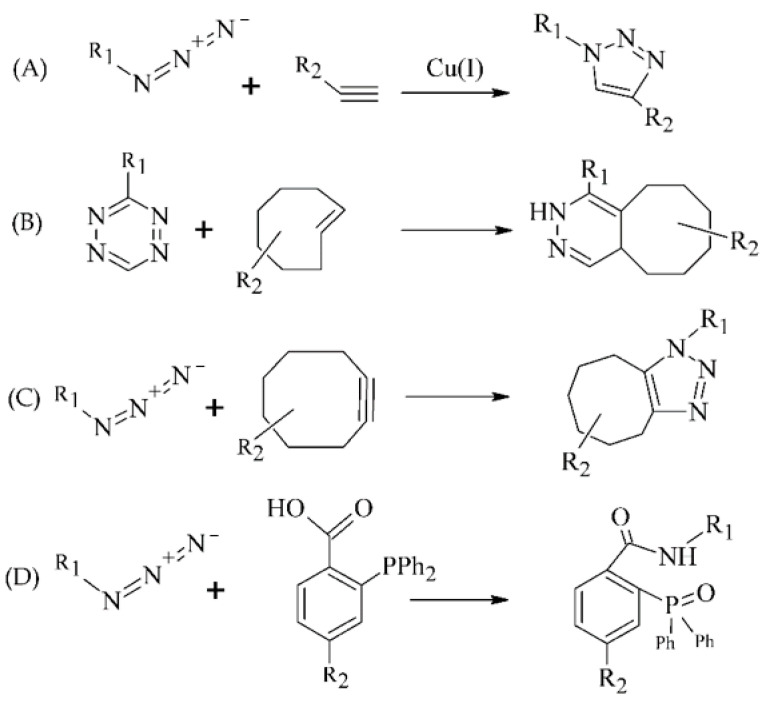
Selected click reactions for the preparation of intermediates used for metal chelating (R_1_, R_2_—proper chelating and peptide moieties). (**A**) CuAAC, (**B**) IEDDA, (**C**) SPAAC, (**D**) Staudinger ligation.

Since Kolb et al. described “click reactions” in 2001 [92], this new chemistry has become rapidly growing in various chemical fields and, since 2006, also in the radiochemistry field. There are two main characteristics making the click chemistry attractive, i.e., the bioorthogonality of reactions and mild reaction conditions (usually at room temperature and in aqueous media) [93]. Additional benefits include the selectivity, rapidity, and modularity of click ligations. The most associated term with the “click chemistry“ is the Cu(I)-catalyzed azide-alkyne cycloaddition (CuAAC) forming 1,4-disubstituted 1,2,3-triazoles (see Figure 7A). Mindt et al. developed and extended the “click-to-chelate” methodology for radiometallic ligation [94,95], in which 1,2,3-triazole is an integral part of the chelating system. This approach has been successfully applied for Tc- and Re-tricarbonyl compounds, when tridentate ligands are coordinated to M(CO)_3_ core resulting in better pharmacokinetic properties [94,95].

In recent years, several catalyst-free site-specific reactions have been investigated for effective radiolabeling of peptide biomolecules and nanomaterials including tetrazines and trans-alkenes for the inverse electron-demand Diels–Alder reaction (IEDDA), azide and cyclooctyne functionalities for the strain-promoted azide-alkyne cycloaddition (SPAAC), and functionalized phosphanes for the Staudinger ligation (Figure 7B–D) [8,96,97]

**Figure 8:**
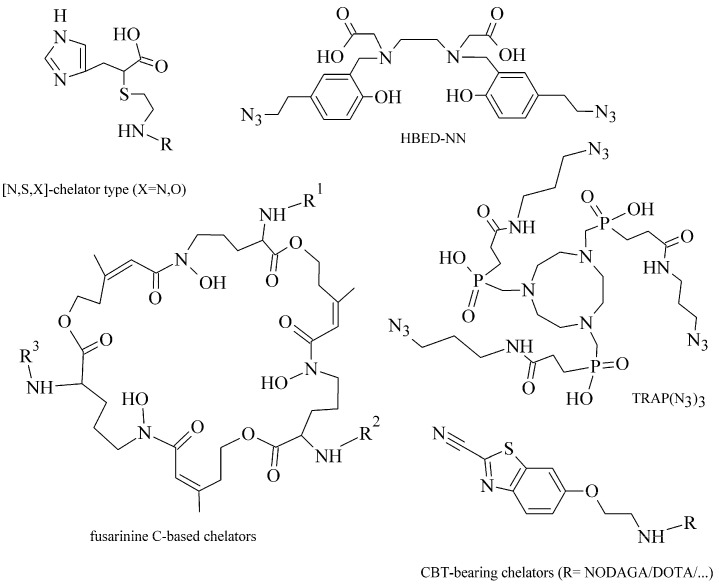
Examples of attractive clickable chelators for radiolabeling of biomolecules with metal radionuclides [106,108,111,112,113].

Within the “click-to-chelate” methodology, the development of new clickable chelators is currently attracting a growing interest (see examples in Figure 8). New clickable chelators have been designed for ^99m^Tc-labeled peptides to obtain an increased hydrophilicity and decreased hepatobiliary retention of ([^99m^Tc]Tc(CO)_3_)-complexes. Novel dipicolylamine derivatives, substituted with carboxylates on the pyridyl rings, were synthesized and evaluated for *fac*-[Tc/Re^I^(CO)_3_]^+^ complexation with α-MSH peptide analogue [98]; a propargyl-substituted thiocarbamoylbenzamidine acting as a tetradentate ligand for a conjugation with [Re/Tc^V^O]^3+^ cores [99]; or 1,4-substituted pyridyl-1,2,3-triazole derivatives with pendent phenyl isothiocyanate groups [100].

For ^68^Ga- and ^64^Cu-labeled probes, standard BFCA have been modified using various prosthetic groups. The DOTA- and NOTA-based click chelators with aldehyde, alkyne, aminooxy, azide, maleimide, monofluorocyclooctyne, and thiol functionalities were developed using CuAAC or RIKEN click reaction [101,102,103,104]; or with azide and tetrazine prosthetic groups using SPAAC and IEDDA reactions [105]. The HBED-chelator was modified with two azide groups (HBED-NN) and both azide and carboxylic groups (HBED-NC) [106]. Novel cyclic hydroxamate siderophore-based BFCA were reported as promising BFCA for gallium-68 [107]. Baranyai et al. optimized a procedure for the conjugation of 1,4,7-triazacyclononane-1,4,7-tris(methylene(2-carboxyethylphosphinic acid)) chelator (TRAP) with peptides using CuAAC [108]. The TRAP conjugates showed kinetic inertness and suitability for ^64^Cu- and ^68^Ga-coordination [109,110].

### 3.4. Radiolabeling Approaches of Peptides with Metallic Radionuclide Based on Nanoparticles

**Figure 9:**
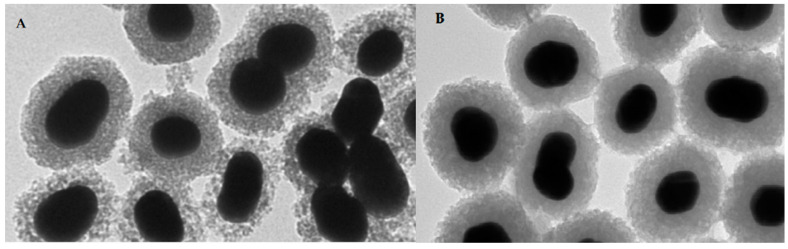
Representative image of PET-SERRS nanoparticles with non-optimized ^68^Ga-labeling (**A**) with visible degradation of silica shells and after the optimization (**B**) with improved stability of the silica shells [118].

Nanomedicine has recently emerged as one of the most promising branches in medicine including a development of novel probes with improved properties for the site-specific detection or therapy of cancer. This rapidly growing trend is underlined by numerous reviews in the radiochemistry field [114,115,116,117]. Over past 10 years, tens of articles have been focused on the metal-labeled nanoparticles (NP) conjugated to various peptides for SPECT and PET cancer imaging (see a representative image of radiolabeled nanoparticles using electron microscopy in Figure 9).

**Figure 10:**
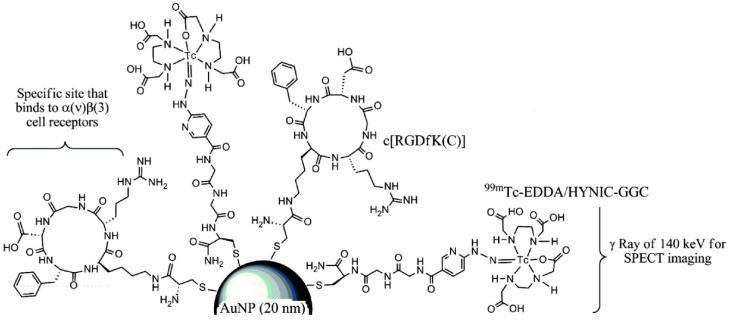
Illustrative scheme of [^99m^Tc]Tc-EDDA/HYNIC-GGC conjugated to RGD derivative and gold NP [119].

Radiolabeling of NPs with technetium-99m can be carried out by a direct or an indirect method. The direct approach is based on a reduction in [^99m^Tc]TcO_4_^−^ with the acidic solution of stannous chloride followed by its direct binding and incorporation to a NP core. In the indirect method, BFCA is necessary to allow a stable linkage between radionuclide and NP [116]. The indirect method has been mostly used for the radiolabeling of ^99m^Tc-NPs conjugated with peptides, see an illustrative example in Figure 10. Gold NPs have been conjugated to peptides with [^99m^Tc]Tc-HYNIC for integrin-positive glioma imaging [119], with [^99m^Tc]Tc-DTPA for breast cancer imaging [120], for gastrin releasing peptide receptor imaging [121,122] and somatostatin receptor-positive neuroendocrine tumor imaging [123]. The NPs based on a polylactic acid polymer were conjugated to ^99m^Tc-labeled octreotide for pancreatic polypeptide-secreting tumor imaging [124].

Several published papers dealt with ^111^In-labeled NPs conjugated to peptides such as directly labeled gold NPs for human melanoma and glioblastoma imaging [125], liposomal NPs conjugated to a RGD peptide analogue and the undecapeptide substance P for glioblastoma and melanoma targeting [126].

Furthermore, ^64^Cu- and ^68^Ga-labeled NPs functionalized with a peptide were reported in several papers too. The multifunctional gold nanorod nanocarriers were covalently bound with doxorubicin and subsequently conjugated to [^64^Cu]Cu-NOTA-RGD [127]; [^64^Cu]Cu-sulphide NPs conjugated to the pegylated bombesin [128]; [^68^Ga]Ga-DOTA-somatostatin and neurotensin analogues to gold NPs [129]; [^68^Ga]Ga-NODAGA-bombesin to the polyethylene glycol-coated ultra-small superparamagnetic iron-oxide nanoparticles [130]; and [^68^Ga]Ga-DOTA-bombesin analogue conjugated to the *N,N,N*-trimethyl chitosan-coated magnetic nanoparticles for a breast cancer detection [131].

## 4. Onco-Receptors and Their Target-Specific Radiometal Labeled Peptide Molecules for Tumor Imaging

**Figure 11:**
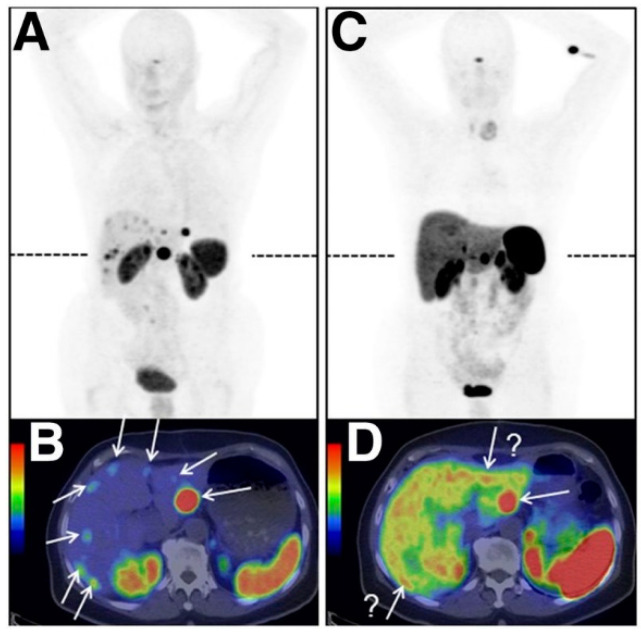
PET/CT images of a patient with ileal neuroendocrine tumors showing bilobar liver metastases (marked with arrows) after application of [^68^Ga]Ga-OPS202 (**A**) and its transaxial fusion image (**B**) and [^68^Ga]Ga-DOTATOC (**C**) and its transaxial fusion image (**D**) (adapted from [60]).

In the following Section 4.1, Section 4.2, Section 4.3, Section 4.4, Section 4.5 and Section 4.6, the most commonly studied onco-receptors are summarized, briefly characterized (location and purpose in human body), and discussed in relation to the development and improvements in their significant radiometal labeled ligands and tumor imaging. In a similar way, radiometal labeled peptide inhibitors of tumor-related proteins (Section 4.7) and sulfonamide-based analogues for tumor hypoxia imaging (Section 4.8) are discussed. In the accompanied tables, examples of particular radiolabeled analogues along with corresponding onco-receptors used in a positive tumor imaging over past three years, advantages and limitations of the studied diagnostic systems are critically evaluated. An illustrative example of a study of radiolabeled [^68^Ga]Ga-OPS202 and [^68^Ga]Ga-DOTATOC biomolecules for NET imaging is in Figure 11.

### 4.1. Somatostatin and Its Analogues for Somatostatin Receptors (SSTR) Imaging

**Figure 12:**
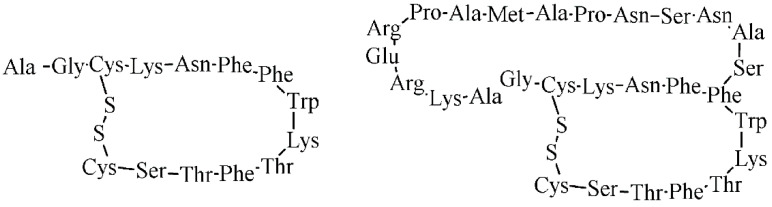
The AA sequence of two biologically active forms of somatostatin.

Somatostatin (SST) is a physiological hormone occurring in two biologically active forms with the AA sequences illustrated in Figure 12. It regulates an endocrine and exocrine secretion throughout a human body.

The biological effects of SST are mediated via 5 types of somatostatin receptors (SSTR) belonging to a G-protein coupled receptors family. SST, its analogues and receptors, have become increasingly popular and widely studied because of anti-tumor effects and mechanisms, including GEP-NETs [132], pituitary adenomas [133], breast cancer [134], small-cell lung cancer [135], melanoma [136], etc. The most commonly expressed receptor subtype in tumor cells is SSTR2, followed by SSTR1, SSTR5, SSTR3, and SSTR4 as the least expressed subtype [137]. Due to short biological half-lives of the natural SST, various synthetic analogues have been designed and evaluated to obtain more stable compounds (see Table 7). It can be stated, based on the examined published papers, there is a great effort to modify the DOTA-octreotide structure in order to achieve novel SST analogues with even better pharmacokinetic properties and specificity to avoid an intense uptake in liver, spleen, and kidney. The SST analogues labeled with gallium-68 and DOTA currently represent the best procedure for GEP-NET imaging. This statement is supported with a large number of research articles that include [^68^Ga]Ga-DOTANOC, DOTATATE, and DOTATOC, respectively, for imaging of various tumors, such as head and neck paraganglioma [138]; pituitary adenoma and meningioma [139]; thyroid [140] and lung [141] carcinoma; and tumors in gastrointestinal system [60] as well. According to available literature from 2010, new approaches for syntheses of the SSTR-ligands seem to be not so extent, but since then, many consecutive examinations and reports have already been comprised of proven ligands for a variety of GEP-NET imaging in clinical trials.

### 4.2. Bombesin and Its Analogues for Gastrin-Releasing Peptide Receptor (GRPR) Imaging

**Figure 13:**
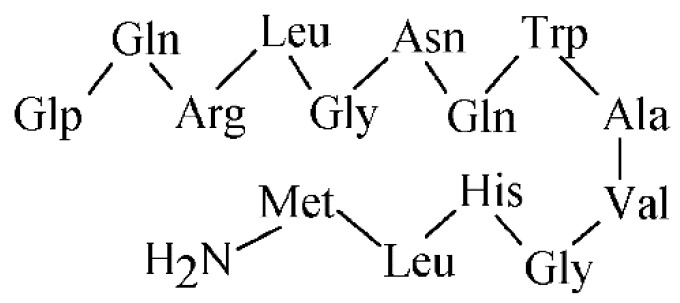
The AA sequence of bombesin peptide.

Bombesin (BBN) is a 14 AA peptide analogue (see the sequence in Figure 13) to the gastrin-releasing peptide and it represents an interesting probe for targeting of gastrin-releasing peptide receptors (GRPR) relevant in oncology.

In total, four receptors belong to the family of GRPR, namely neuromendin B receptor BBR_1_, gastrin-releasing peptide receptor BBR_2_, orphan receptor BBR_3_, and amphibious receptor BBR_4_. Predominantly the BBR_2_ is upregulated in cancer cells such as breast, lung, pancreas, colon, and prostate [149]. Research with radiolabeled BBN analogues has become increasing since the development of [^99m^Tc]Tc-Lys^3^-BBN in 1998 [150]. Since then, most of these radiolabeled analogues have been designed as GRPR agonists with a favorable internalization in cancer cells. Meanwhile, several studies have demonstrated unwanted side effects of agonists connected with their GRPR activation, thus a research field has shifted its interest to antagonists [151]. Radiolabeled GRPR antagonists have shown superior value to the agonists in terms of better pharmacokinetic properties, very good in vivo stability and, by that, sufficient retention in cancer cells [152]. New GRPR antagonists have been developed with a potential for the clinical translations (see summarized studies in Table 8).

### 4.3. Cholecystokinin and Its Analogues for Cholecystokinin Receptor (CCKR) Imaging

**Figure 14:**
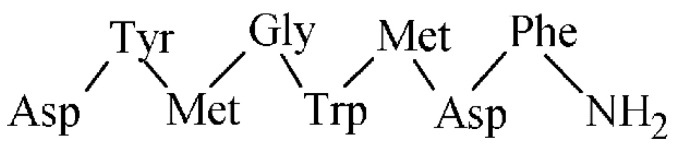
The AA sequence of octapeptide cholecystokinin.

Cholecystokinin (CCK) is a peptide hormone, which regulates various actions predominantly in the gastrointestinal tract and central nervous system. CCK was initially characterized with a 33 AA sequence, but later, the peptide was shown to be present in more biologically active forms (e.g., CCK4, CCK8, CCK33, CCK39) derived from a 115 AA precursor [159]. A total of three types of CCK receptors from the G-protein coupled receptors family have been identified, CCK1 known as CCK A, CCK2 known as CCK B, and CCK2i4sv receptor, respectively. The extensively studied receptors are CCK1, characterized in pancreatic cells and mainly located in periphery, and CCK2 located in the brain, stomach, pancreas, and gall bladder, and overexpressed in cancer types such as small cell lung cancers and medullary thyroid carcinomas [159]. The cholecystokinin octapeptide CCK8 (see its AA sequence in Figure 14) and minigastrin are of the most evaluated molecules for CCK2 receptors. All synthesized peptide analogues have the C-terminal receptor-binding tetrapeptide sequence of Trp-Met-Asp-Phe-NH_2_ in common. Many of the CCK8 and minigastrin analogues were developed and evaluated up to 2010, the studies over past 3 years are summarized in Table 9.

### 4.4. Exendin Analogues for Glucagon-Like Peptide 1 (GLP-1) Receptor Imaging

**Figure 15:**
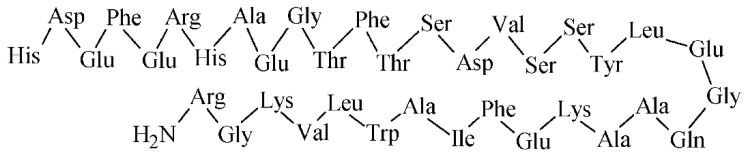
The AA sequence of GLP-1.

Glucagon-like peptide 1 (GLP-1) is an intestinal peptide hormone with a 36 AA sequence (see Figure 15), which stimulates insulin secretion. An action of the GLP-1 and its analogues is mediated through a glucagon-like peptide-1 receptor as a class B of G-protein-coupled receptor. The GLP-1 receptor was identified by radioligand binding experiments [164] and is expressed mainly in the stomach, pancreas, and brain. The GLP-1 receptor has been found predominantly in insulinomas, gastrinoma, pulmonary neuroendocrine tumors, and medullary thyroid cancer. GLP-1 analogues have been synthesized for the GLP-1 receptor targeting, from which exendin-4 as an agonist and exendin-3 as an antagonist have been widely studied (Table 10).

### 4.5. RGD Analogues for Integrin Receptors Imaging

**Figure 16:**
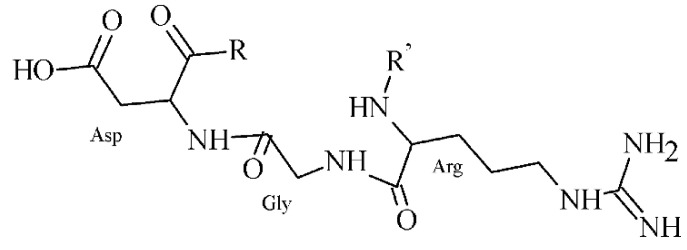
Structure of a peptide containing RGD sequence.

Nowadays, over 20 subtypes of integrin family receptors are known, from which α_v_β_3_, but also α_v_β_5_ and α_v_β_6_ are of well-studied subtypes recognizing the Arg-Gly-Asp (RGD) peptide (Figure 16), and their expression correlates with metastasis.

An enhanced α_v_β_3_ expression is associated with angiogenesis, tumor growth, invasion, and metastasis. The α_v_β_3_ integrins expression has been demonstrated in various endothelial and cancer cells such as breast, gastric, non-small cell lung, pancreatic, ovarian, and prostate cancer, oral squamous cell carcinoma, melanoma, or glioma [167]. Over the last decades, many radiolabeled bioactive molecules with the RGD motif have been synthesized and evaluated for the integrin α_v_β_3_-positive tumors targeting, providing useful conjugates for clinical translation (see summary in Table 11). Since 2018, a number of traditional syntheses of novel BFCA-RGD conjugates has rapidly decreased due to the utilization of RGD peptides for a nanoparticle coupling.

### 4.6. Other Radiometal Labeled Peptide Analogues for Imaging of Other Tumor Receptors

**Figure 17:**
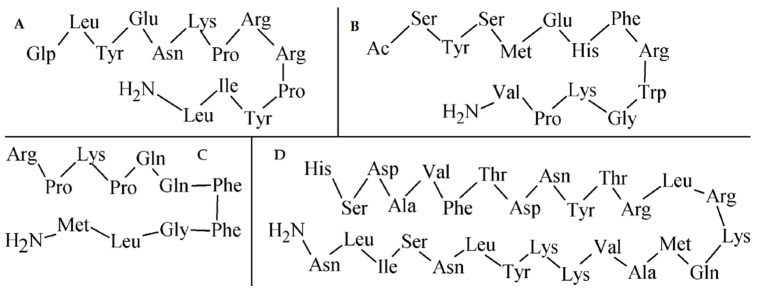
AA sequences of other important peptides for tumor imaging. (**A**) neurotensin; (**B**) α-MSH; (**C**) substance P; (**D**) VIP.

Neurotensin (NT), α-melanocyte stimulating hormone (α-MSH), substance P, and vasoactive intestinal peptide (VIP) represent other important radiometal labeled peptide analogues for imaging of various other significant tumor receptors (Figure 17).

The NT is a neurotransmitter and hormone with a sequence of 13 AA, in which the C-terminal NT(8–13) is responsible for affinity and activity to a NT receptor. There are three types of the NT receptors: NTR_1_–NTR_3_, where NTR_1_ is an extensively studied receptor and a promising target for cancer imaging. The NTR_1_ overexpression has been demonstrated in a tumor progression, e.g., in pancreas and colon adenoma, but also in breast, lung, or prostate cancer, while the expression of NTR_2_ has been reported in prostate cancer, lymphatic leukemia, and glioma [175]. Several NT analogues have been developed as effective targets for colorectal adenocarcinoma cells (Table 12).

The α-MSH is a neuropeptide with a sequence of 13 AA that is selectively bound to a melanocortine-1 receptor (MC1) overexpressed in leukocytes, melanocytes, and transformed melanoma cells, and is primarily responsible for a regulation of inflammatory state and skin pigmentation [176]. Numerous α-MSH analogues have been developed as attractive targets for melanoma radiodiagnosis or imaging (Table 12).

The substance P with a 11-AA sequence belongs to a family of tachykinins and exerts its activity through the G protein-coupled neurokinin receptors (NKR), i.e., NK_1_R–NK_3_R, with the highest affinity of NK_1_R. The substance P has been found in various cell systems bearing NK_1_R, such as immune cells, monocytes, macrophages, lymphocytes, microglia, dendritic cells, bone marrow stem cells, and others. In the central nervous system, NK_1_R are expressed in neurons, astrocytes, microglia, and cerebral endothelial cells [177]. Effects of the substance P in human organism include: immune and secretion stimulation, smooth muscle contraction (pulmonary, urinary, GIT, and vascular system), and is involved also in a pain transmission, vasodilatation, connective-tissue cell proliferation, and neuroimmune modulation [177]. Thus, substance P analogues and NK_1_R antagonists have been synthesized and used for the NK_1_R-positive tumor detection as shown in Table 12.

The VIP is a peptide with a 28 AA sequence that regulates various immune cells, promotes vasodilatation, growth and function of tumor cells. Its biological action is mediated through three classes of the G-protein-coupled receptors VPAC1, VPAC2, and PAC1. The receptors for VIP occurs in numerous tumor cells including thyroid, breast, lung, liver, pancreas, intestinal epithelial cells, colon, bladder, prostate, uterus, and neuroendocrine tumors [178,179].

### 4.7. Small Peptide Inhibitors of Proteins for Protein-Positive Tumor Imaging

Many protein interactions in a biological system are responsible for an origination or progression of various diseases including cancer. In recent years, inhibitors of such proteins based on small peptide biomolecules are widely developed and investigated. This subsection covers the latest radiolabeled peptide inhibitors of the prostate-specific membrane antigen (PSMA) and fibroblast activation protein (FAP) for imaging of related tumors (see summarized studies in Table 13).

**Figure 18:**
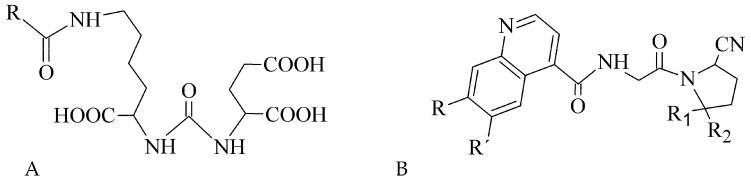
Structural motifs of small peptide inhibitors of proteins. (**A**) EuK motif as a base for PSMA inhibitors, (**B**) 2-cyanopyrrolidin-quinoline carboxamides as a base for FAP inhibitors.

The PSMA is a membrane-bound folate gamma glutamyl-carboxypeptidase II, which is physiologically present in various tissues, e.g., salivary glands, ovary, prostate epithelium, and astrocytes [191]. From the cancerous cells, it is primarily expressed in benign and malignant prostatic tissue [192]. However, studies on the PSMA-expression in also other tumor types are available, including breast, gastric, and colorectal cancer, lung and renal carcinoma, and brain tumors [193,194,195,196,197,198]. Thus, PSMA has become one of the most promising and extensively evaluated molecular targets in nuclear medicine. Research was mainly focused on monoclonal antibodies, but various radiolabeled small peptide-based inhibitors containing Glu-C(O)-Lys (EuK) sequence (see Figure 18A) have been recently developed to effectively localize and treat related tumors. Other two functionalities, i.e., phosphonates and thiols, with affinity to PSMA have been identified. The most widely used example of such inhibitor is the [^68^Ga]Ga-PSMA-11 (i.e., ^68^Ga-labeled Glu-NH-CO-NH-Lys(Ahx)-HBED-CC) [199]. At present, it is included in many clinical trials that monitor various conditions in a prostate cancer management. 

Another extensively studied protein with selective expression in several tumor types is FAP, a serine protease. The FAP protein has been associated with fibrosis, inflammation and cancer, and is undetectable in a majority of normal adult tissues [200]. Several works revealed its localization not only in activated fibroblasts [201], but also in endothelial cells and macrophages [202,203]. The participation of FAP in a cell invasiveness, proliferation, migration and tumor vascularization has been described [204]. The FAP overexpression and activation has been observed in various malignancies, e.g., pancreatic, hepatocellular, lung, breast, colorectal, or ovarian [205,206,207,208,209,210]. Different strategies are investigated to target FAP activity such as (i) probes with fluorescent moiety, (ii) prodrug delivery systems, (iii) FAP inhibitors (FAPI), and (iv) immune-based pathways [211]. Radiolabeled peptide FAPI based on 2-cyanopyrrolidin-quinoline carboxamide structure (Figure 18B) were developed [212] and then FAPI linkers have been modified to improve pharmacokinetic properties, tumor binding, and PET images [213]. Further structural modifications and clinical studies are underway and thus FAPI represent new attractive imaging and therapeutic options for oncological diseases.

### 4.8. Radiometal Labeled Sulfonamide-Based Analogues for Tumor Hypoxia Imaging

**Figure 19:**
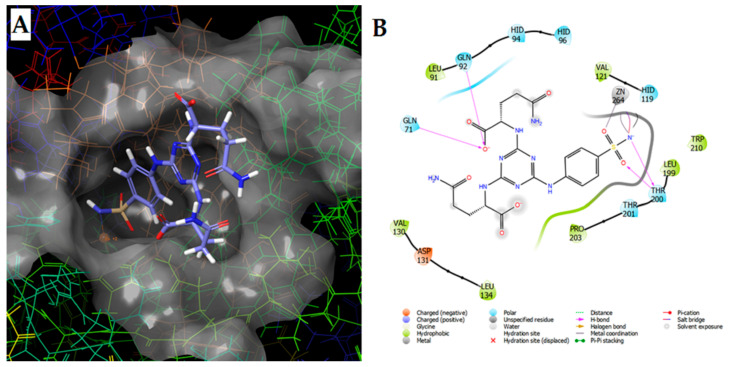
Position of sulfonamide-derived hCA IX inhibitor docked into the active site of hCA IX (**A**) and its intermolecular interaction diagram (**B**) [239].

Hypoxia, a phenomenon when a level of oxygen is below its demands, is a common feature for tumor development and progression. Many solid tumors have regions permanently or transiently exposed to hypoxia because of aberrant vascularization and a poor blood supply [231]. Since hypoxia is a key component in cellular expression, tumor blood vessel formation, cancer progression, metastasis, often leading to cell death, a current research in this area is focused to an early detection and selective monitoring or suppression of hypoxic tissues to effectively minimize all possible complications associated with this phenomenon. Many studies have been comprised of radiolabeled small nitroimidazole derivatives [232,233,234,235], and monoclonal antibodies [236,237], resulting in a development of new agents capable of accessing to overexpressed proteins under hypoxic state (i.e., hypoxia inducible factor HIF-1 regulated genes for carbonic anhydrase CA IX, vascular endothelial growth factor, angiopoietin-2, etc. [238]). Nevertheless, small sulfonamide- and peptide-based biomolecules labeled with metal radionuclides have been studied for imaging of various hypoxic tumor cells overexpressing CA IX as one of the prominent gene in the HIF-induced processes (see summary in Table 14). A highly specific binding of various sulfonamide derivatives with amino-acid substituents has been demonstrated in our several recent works. For example, an illustrative superposition and intermolecular interaction diagram of potential 1,3,5-triazinyl-sulfonamide inhibitor docked into the active site of human CA IX are in Figure 19A,B.

## 5. Concluding Remarks and Future Perspectives

Various chemical types of metallic radiopharmaceuticals for use in oncology are approved by the European Medicines Agency or U.S. Food and Drug Administration. Apart from these registered radioactive medicines, a much larger scale of radiolabeled bioactive ligands is under investigation in nuclear research or clinical trials. In this review, recent advances in the radiolabeling process of amino-acid based biomolecules, the most commonly used metal radionuclides, their chemistry and BFCA, as well as the most important peptide receptor families (including currently the most perspective field of PSMA and FAP ligands), were critically discussed. Continual efforts in proposing new structures with improved pharmacokinetic properties for selective targeting of cancer cells and effective utilization in imaging techniques should be guaranteed. The disease imaging on a molecular level, as well as radionuclide availability on-site, lower radiation burden, detection of early stage problem, and monitoring of a response to treatment in the combination with targeted therapy for a personalized approach to a patient, have a great potential to bring additional valuable outputs in the field of nuclear medicine in future.

Over the past years, great progress in a radiolabeling with metallic radionuclides has been demonstrated, owing to a development of many new chelators (or new derivatives of well-known traditional chelators) and linkers for an effective connection between metals and biomolecules. Modern chelators such as TRAP, THP, and FSC for gallium-68, DFO for zirconium-89, sarcophagines for copper-64, tricarbonyl and [N,S,X]-type chelators for technetium-99m and their modifications have been designed to improve binding affinity and pharmacokinetic properties of a radiolabeled probe for its molecular target. In spite of remarkable progress, there is still an enormous need to develop target specific compounds with improved pharmacokinetics and selectivity to a desired in vivo target, because many studies have confirmed various complications in the development. These are mainly lower stability, higher toxicity, adverse pharmacokinetic behavior, and higher retention of radioactivity in studied material in vivo and in vitro. In this context, amino acid moieties proved to be ones of the most suitable linkers to complete a target-specific structure. Optimized structures of some of the newly developed radiolabeled biomolecules should provide enhanced affinity and selectivity to the onco-receptors, lower radiation dosage for patient, decreased interactions with other drugs or physiological proteins, without misrepresenting results, and, by that, a more favorable utilization in diagnostic nuclear medicine over other imaging techniques (e.g., MRI, CT).

Peptides, as amino acid based biomolecules, represent current and future important tools in a development of target-specific radiolabeled compounds. It is due to a high degree of their compatibility with many protein structures overexpressed in various diseases, including cancer, as the second leading cause of death globally. Current research, with a promising perspective, is directed mainly towards peptide radiolabeled agents that are aimed at proteins overexpressed in pancreatic, colorectal, prostate, and brain tumors. These types belong to the most frequently diagnosed and the most severe cancers. The integrin α_v_β_3_ receptors from traditional receptor families and PSMA, as well as FAP ligands are very attractive and perspective probes due to their intense association and overexpression within a variety of cancer cells and new vasculature in general, and so tumor growth, proliferation, and metastasis.

As emerged from the reviewed studies dealing with an implementation of imaging methods (PET, SPECT, etc.), in nuclear medicine research, gallium-68, DOTA-based chelators, and amino acid linkers are currently dominating in the research of new potential diagnostic and imaging agents. In centers, where ^68^Ga-compounds cannot be used due to gallium unavailability, alternative PET labels were introduced. For example, yttrium-86 or zirconium-89 could be employed since a remarkable development in small medical cyclotrons has been achieved over past years. However, there are still new ^99m^Tc-labeled analogues for SPECT imaging as an alternative method of PET tracers. Other interesting non-standard radionuclides such as cobalt-55, scandium-44, titanium-45, and manganese-52 are increasingly utilized in preclinical studies and could be a merit of future investigations in clinical field. These non-standard metal radionuclides with their therapeutic pairs represent the highly attractive labels for development of theranostic approaches as precise predictive biomarkers of a response to therapy strategies. The inherent part of a diagnostic or imaging process is an applied imaging technique. It is evident that hybrid methods of SPECT and PET combined with CT is of routine. The ongoing studies could be focused on a development of probes and methodologies with high anatomical and functional sensitivity, spatial resolution, as well as mentioned superior pharmacokinetic profile for a better disease management using SPECT and PET with MRI as an important tool to improve the diagnostics, staging and planning of treatment strategy.

## Figures and Tables

**Table 1:** Selected metallic radionuclides employed in diagnostic nuclear medicine.

Isotope	Radiation Type	E_max_ [keV] (Decay %)	Half-Life	Production (Common Reaction)	Main Application Areas
^99m^Tc	γ	141 (89.1%)	6.01 h	^99^Mo/^99m^Tc generator (cyclotron alternatively)	SPECT of lung, brain, myocard, bones, kidney, liver, etc.
^111^In		171.3 (90.2%) 245.4 (94%)	2.83 d	cyclotron, ^112^Cd(p, 2n)^111^In	SPECT of somatostatin receptor-positive NET
^67^Ga		93.3 (37%), 184.6 (20.4%), 300.2 (16.6%)	3.26 d	cyclotron, ^68^Zn(p, 2n)^67^Ga	scintigraphy of inflammation, infection, tumors
^64^Cu	β^+^	653 (17.6%)	12.7 h	cyclotron, ^64^Ni(p, n)^64^Cu	PET imaging of hypoxic tumors, integrin- and gastrin-releasing peptide receptor-positive tumors
^68^Ga		836 (89%)	67.7 m	^68^Ge/^68^Ga generator (cyclotron alternatively)	PET imaging of somatostatin receptor-, PSMA-, FAP-overexpressed tumors
^89^Zr		395 (23%)	3.3 d	cyclotron, ^89^Y(p, n)^89^Zr	immuno-PET imaging

Dosimetry and imaging aspects, depending on a particular radiolabeled compound and its properties, as well as an overall condition of a patient, can be found (if they were evaluated) in individual imaging studies discussed in Section 4.

**Table 2:** The most common BFCA for ^99m^Tc-labeled compounds.

BFCA	Complex Structure	References
DTPA and its derivatives	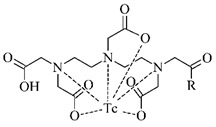	[46]
MAG3	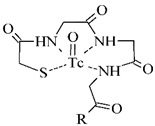	[47]
HYNIC (with tricine coligand)	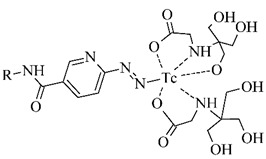	[48]

**Table 3:** The most common BFCA for ^68^Ga-labeled compounds.

BFCA	Complex Structure	References
DOTA and its derivatives	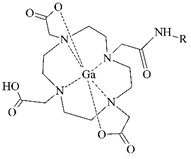	[21,50,51]
NOTA and its derivatives	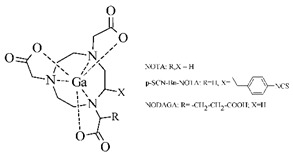	[52]
AAZTA and its derivatives	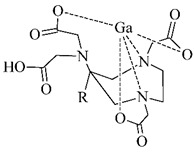	[53]
HBED-CC	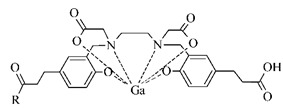	[54,55]
PrP9 and its derivatives	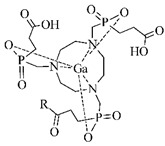	[56]
THP and its derivatives	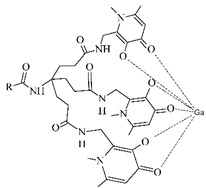	[57]

**Table 4:** The most common BFCA for ^111^In-labeled compounds.

BFCA	Complex Structure	References
DOTA and its derivatives	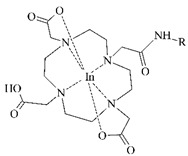	[64,65]
DTPA and its derivatives	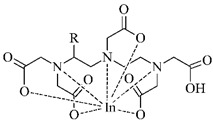	[66]

**Table 5:** The most common BFCA for ^64^Cu-labeled compounds.

BFCA	Complex Structure	References
NOTA and its derivatives	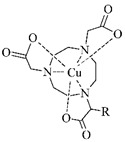	[78,79]
DOTA and its derivatives	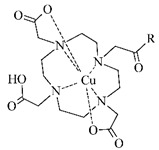	[80]
bisthiosemicarbazones	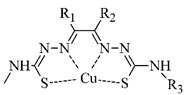	[24,81,82]
cyclam	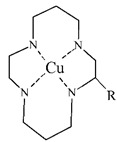	[26]
TETA and its derivatives	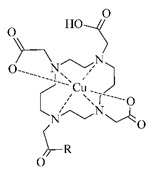	[83,84]
sarcophagines	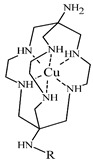	[28,85]

**Table 6:** The most common BFCA for ^89^Zr-labeled compounds.

BFCA	Complex Structure	References
DFO and its derivatives	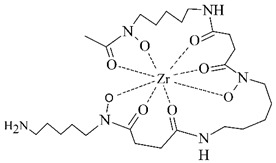	[89]
2,3-HOPO and its derivatives	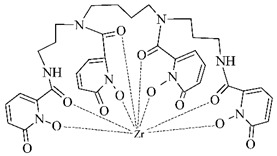	[90]
DTPA and its derivatives	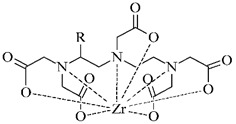	[91]

**Table 7:** Summary of radiolabeled somatostatin analogues for SSTR-positive tumor imaging over past 3 years.

Composition of Studied Compounds - Metal Radionuclide - BFCA - Linker - Peptide	Results and Findings - Phase of Trials - Cancer Type Studied - Imaging Technique Used - Benefits/Limitations/Conclusion	Reference
- ^68^Ga - DOTA - x - TOC, TATE	- clinical, 10 patients - metastatic NET - PET, PET/CT - reduced signal from the liver achieved; methodology improvement needed for implementation of parametric-based kinetic analysis	[142]
- ^99m^Tc, ^177^Lu - DOTA, HYNIC, EDDA -6-carboxy-1,4,8,11-tetraazaundecane (N4) -p-Cl-Phe-cyclo(d-Cys-Tyr-d-Trp-Lys-Thr-Cys)d-Tyr-NH_2_	- preclinical in vitro, in vivo - kidneys - SPECT/CT - more useful biodistribution results for a highly potent [^99m^Tc]Tc-N4-conjugate than with lutetium-177; HYNIC-conjugate with complete loss of SSTR-2 affinity	[143]
- ^68^Ga - DOTA, NODAGA - x - JR11, TOC	- clinical, 12 patients - GEP-NET - PET/CT - very high TBR and image contrast of liver lesions for [^68^Ga]Ga-NODAGA-JR11; studies in larger patient group proven	[60]
- ^68^Ga - DOTA, fluorescein isothiocyanate - x -PA1, TATE	- preclinical in vitro, in vivo - lung, colorectal and gastric - microPET - effective tumor targeting and lower kidney accumulation of [^68^Ga]Ga-DOTA-PA1; a potential for PET/CT of SSTR-positive tumors (especially lung) suggested	[144]
- ^64^Cu - NODAGA, DOTA - x - JR11, TATE	- preclinical in vitro, in vivo - kidneys - microPET - more favorable in vivo pharmacokinetics, low levels in the liver, spleen and rapid blood clearance for [^64^Cu]Cu-NODAGA-JR11 with further development for clinical translation	[145]
- ^99m^Tc - HYNIC, EDDA, tricine - x - TATE	- preclinical in vitro, in vivo; clinical, 6 patients - NET - SPECT/CT - reproducible kit with 2.96 GBq/6 mL formulated, but some differences in tumor uptake occurred	[146]
- ^68^Ga - DOTA - x - TOC	- clinical, 4 patients - GEP-NET - PET/MRI - sensitive and accurate evaluation of the liver, but limited accuracy of MRI related to lung and bone diseases	[147]
- ^68^Ga - DATA - x - TOC	- clinical, 53 patients - GEP-NET - PET/CT - comparable imaging profile of [^68^Ga]Ga-DATA-TOC with DOTA-NOC; DATA-conjugate useful for instant kit labeling	[148]

**Table 8:** Summary of radiolabeled bombesin analogues for GRPR-positive tumor imaging over past 3 years.

Composition of Studied Compounds - Metal Radionuclide - BFCA - Linker - Peptide	Results and Findings - Phase of Trials - Cancer Type Studied - Imaging Technique Used - Benefits/Limitations/Conclusion	Reference
- ^67^Ga, ^68^Ga, ^111^In, ^177^Lu - DOTA - *p*-aminomethylaniline-diglycolic acid - NeoBOMB1	- preclinical in vitro, in vivo; clinical, 4 patients - prostate - PET/CT - [^68^Ga]Ga-NeoBOMB1 with preserved GRPR affinity, high in vivo stability, and high contrast image in patients	[152]
- ^55^Co, ^57^Co - NOTA - PEG_2_ - RM26	- preclinical in vitro, in vivo - prostate - SPECT/CT, PET/CT - favorable pharmacokinetics and 3-fold lower internalization of ^55^Co-labeled peptide compared to ^111^In-labeled conjugate making it potential “next day” high contrast PET imaging probe	[153]
- ^64^Cu - DOTA with hydroxamic acid arms (DOTHA_2_), NOTA - PEG - RM26	- preclinical in vitro, in vivo - prostate - microPET/CT - fast elimination and slightly better in vivo imaging properties for DOTHA_2_-conjugate than reference	[73]
- ^64^Cu - DOTA, NODAGA - [Pro-Gly]_12_ linker, PEG_3_ - RGD, BBN(7–14)	- preclinical in vitro, in vivo - prostate - microPET - NODAGA-conjugate for dual α_v_β_3_/GRPR targeting with better pharmacokinetics than DOTA, but low tumor uptake in vivo	[74]
- ^68^Ga - DOTA - *N*-(γ-maleimidobutyryloxy) succinimide ester - PSMA, Lys^3^-BBN(1-14)	- preclinical in vitro, in vivo - pulmonary and prostate - microPET/CT - higher cell uptake and internalization, greater affinity for GRPR but lower for PSMA of dimer compared to single [^68^Ga]Ga-BBN/-PSMA monomers	[154]
- ^68^Ga - DOTA -4-amino-1-carboxymethylpiperidine - RM2	- clinical, 16 patients - prostate - PET/CT, multiparametric MRI - fusion of MRI and PET/CT improved detection of a primary disease, but expression of GRPR and PSMA was not correlated	[155]
- ^68^Ga - DOTA prepared from cyclen, DOTA-tris-(t-Bu) ester - x - BBN derivatives	- preclinical in vitro, in vivo - breast and prostate - preclinical nanoPET/CT - potency and efficiency of site-specific DOTA-cyclen comparable to that of DOTA-ester	[156]
- ^99m^Tc - N4-chelator - PEG_2-4_ -D-Phe-Gln-Trp-Ala-Val-Gly-His-Leu-NH-CH_2_-CH_3_	- preclinical in vitro, in vivo - prostate - gamma counter - PEG spacer length with only little effect on GRPR affinity, tumor uptake and in vivo stability	[157]
- ^44g^Sc, ^68^Ga - DOTA - aminovaleric acid -Gln^7^-Trp^8^-Ala^9^-Val^10^-Sar^11^-His^12^-FA01010^13^-Tle^14^-NH_2_	- preclinical in vitro, in vivo - prostate and breast cancer - PET/CT - ^44g^Sc-conjugate with low uptake in breast cancer cells, but high tumor uptake and retention in prostate; differences in in vitro GRPR binding properties, but no in in vivo	[158]

**Table 9:** Summary of radiolabeled CCK/minigastrin analogues for CCKR-positive tumor imaging over past 3 years.

Composition of Studied Compounds - Metal Radionuclide - BFCA - Linker - Peptide	Results and Findings - Phase of Trials - Cancer Type Studied - Imaging Technique Used - Benefits/Limitations/Conclusion	Reference
- ^68^Ga, ^89^Zr - fusarinine C (FSC) - x -MG11	- preclinical in vitro, in vivo - epidermoid - microPET/CT - decreased hydrophilicity, increased metabolic stability and kidney retention for dimer and trimer, and reduced TBR of ^89^Zr-monomer and dimers	[160]
- ^111^In - DOTA - x - minigastrins MGS1, MGS2, MGS3, MGS4	- preclinical in vitro, in vivo - epidermoid, pancreatic - nanoSPECT/CT - modified C-terminal of [^111^In]In-DOTA-MGS4 led to high CCK2R affinity, an improved biodistribution profile and a promising in vivo stability, tumor targeting, and TBR	[161]
- ^99m^Tc - HYNIC, EDDA - x -MGS5, MGS11	- preclinical in vitro, in vivo - epidermoid - gamma counter, autoradiography - [^99m^Tc]Tc-HYNIC-MGS11 with high resistance against enzymatic degradation and useful targeting profile similar to DOTA-analogue; a promising kit development of for CCK2R-imaging and radioguided surgery	[162]
- ^111^In - DOTA - x - (D-Glu^1−6^)minigastrin	- clinical, 16 patients - advanced medullary thyroid - SPECT/CT - high uptake in lesions and favorable dosimetry confirmed, but increased calcitonin concentrations in blood; initiation of ^177^Lu-analogue assessment	[163]

**Table 10:** Summary of radiolabeled exendin analogues for GLP-1 receptor-positive tumor imaging over past 3 years.

Composition of Studied Compounds - Metal Radionuclide - BFCA - Linker - Peptide	Results and Findings - Phase of Trials - Cancer Type Studied - Imaging Technique Used - Benefits/Limitations/Conclusion	Reference
- ^111^In - DTPA - lysine - exendin-3	- preclinical in vitro, in vivo - insulinoma - SPECT - hexendin^40–45^ conjugate (with 6 Lys and 6 DTPA residues) as the most useful due to the 7-fold higher specific activity than simpler conjugates and improved visualization of the pancreas	[67]
- ^111^In - NODAGA - albumin-binding moiety (ABM) - exendin-4	- preclinical in vitro, in vivo - insulinoma - SPECT/CT - significantly reduced kidney uptake and improved GLP1R targeting, but a further assessment of whole-body doses needed	[68]
- ^68^Ga - NOTA - methylaminolevulinate - Cys^39^-exendin-4	- preclinical in vitro, in vivo - pheochromocytoma (PCM) - microPET - specific GLP1R targeting in both poorly and highly differentiated PCM cells, but high accumulation in kidneys; more studies needed to establish association between GLP-1R PET and a risk stratification of PCM	[165]
- ^64^Cu - NODAGA x - Lys^40^-exendin-4	- preclinical in vivo - insulinoma - PET/MRI - high background signal from the exocrine pancreas observed during an early time points; the positive correlation between [^64^Cu]Cu-Ex4, reflecting β-cell mass, and Mn-retention demonstrated by a simultaneous PET/MRI	[166]

**Table 11:** Summary of radiolabeled RGD analogues for α_v_β_3_ receptor-positive tumor imaging over past 3 years.

Composition of Studied Compounds - Metal Radionuclide - BFCA - Linker - Peptide	Results and Findings - Phase of Trials - Cancer Type Studied - Imaging Technique Used - Benefits/Limitations/Conclusion	Reference
- ^68^Ga - DOTA, TRAP, FSC, THP - glutamic acid - (RGD)_3_, [c(RGDfK)]_2_	- preclinical in vitro, in vivo - renal, head and neck - microPET/CT - the highest tumor uptake for FSC- and THP-conjugates, but further studies on binding behavior to integrins needed	[168]
- ^99m^Tc - glucoheptonate, D-penicillamine - Ahx - c(RGDfK)	- preclinical in vitro, in vivo - glioma - microSPECT/CT - [^99m^Tc]Tc-[Pen-Ahx-c(RGDfK)]_2_ with the 10-fold higher integrin affinity than the monovalent (Pen-Ahx-c(RGDfK)), but high uptake in the liver, intestine, and kidney calls for an improvement of pharmacokinetics	[169]
- ^99m^Tc - IDA - aspartic acid - [c(RGDfK)]_2_	- clinical, 6 patients - x - gamma camera - radiation doses of renal and biliary system comparable to other ^99m^Tc-labeled peptides, further dosimetry studies needed for a risk-benefit assessment	[170]
- ^68^Ga - NOTA-NHS - 6-Ahx, cysteine - c(RGDyK), GE11	- preclinical in vitro, in vivo - lung - PET/CT - enhanced tumor accumulation of [^68^Ga]Ga-NOTA-RGD-GE11 than monomeric RGD-conjugate, but a modification of linkers needed for an improvement of pharmacokinetics	[171]
- ^68^Ga - NOTA - PEG3, symmetric β-glutamate linker - RGD2	- preclinical in vitro, in vivo - prostate - PET - significant limitations due to high renal and bladder accumulation, but low uptake in other organs	[172]
- ^99m^Tc - HYNIC, tricine, TPPTS - x - RGD2	- clinical, 20 patients - breast - gamma camera - good uptake in breast lesions and also metastatic sites in lymph nodes visible in 2 patients - useful easily available kit for further clinical studies	[173]
- ^68^Ga - DOTA - glutamic acid - (cRGDfK)2	- preclinical in vitro, in vivo - lung - PET/CT - ^68^Ga-labeled conjugate with highly hydrophilic properties, high tumor accumulation, moderate in vivo uptake in kidneys and intestine, with a potential for early detection of lung lesions	[174]

**Table 12:** Summary of radiolabeled NT, α-MSH, substance P, and VIP analogues for other important receptor-positive tumor imaging over past 3 years.

Composition of Studied Compounds - Metal Radionuclide - BFCA - Linker - Peptide	Results and Findings - Phase of Trials - Cancer Type Studied - Imaging Technique Used - Benefits/Limitations/Conclusion	Reference
- ^99m^Tc - HYNIC, EDDA, tricine - x - [Ac-Lys^5^, Pro^6^, βAla^7^, Tle^12^]NT(5–13)	- preclinical in vitro, in vivo - colorectal - gamma camera - useful for early tumor SPECT staging due to appropriate tumor accumulation, high stability, low liver accumulation, and high kidney excretion	[180]
- ^68^Ga - DOTA(*t*Bu)_3_ - 4- amino piperidin-1-yl-acetic acid - Lys^8^-Lys^9^-Pro^10^-Tyr^11^-Ile^12^-Leu^13^-OH modified with TMSAla^12/13^	- preclinical in vitro, in vivo - colorectal - microPET/CT - good NTR1 selectivity and prolonged plasmatic half-life of [^68^Ga]Ga-(TMSAla^13^)-conjugate; further in vivo uptake and impact of other metals (^111^In, ^177^Lu, ^161^Tb) under investigation	[181]
- ^99m^Tc - DPA - Ahx-βAla, ethylene glycol (EG) based linker -Nle-Asp-His-D-Phe-Arg-Trp-Gly-NH_2_	- preclinical in vitro, initial in vivo - melanoma - x - high in vitro stability of [^99m^Tc]Tc-tricarbonyl-DPA base; EG linker more useful than Ahx	[182]
- ^111^In - NHS-DOTA (3-arm), SCN-Bn-DOTA (4-arm) - x - α-MSH	- preclinical in vitro, in vivo - melanoma - SPECT - higher lipophilicity, higher MC1-R affinity, and relatively higher stability of 4-arm DOTA-conjugates than 3-arm	[183]
- ^64^Cu -SCN-NOTA, bispidine carbonate, SCN-dipyridylmethyl-TACN - Ahx-β-Ala -Nle-Asp-His-D-Phe-Arg-Trp-Gly-NH_2_	- preclinical in vitro, initial in vivo - melanoma - gamma counter - high hydrophilicity and sufficient MC1R-affinity of ^64^Cu-conjugate, but lower than that of [^125^I]I-NDP-MSH	[184]
- ^99m^Tc - NOTA, NODAGA - Gly-Gly-Nle -c[Asp-His-DPhe-Arg-Trp-Lys]-NH_2_	- preclinical in vitro, in vivo - melanoma - nanoSPECT/CT - NOTA-conjugate with better tumor targeting and biodistribution properties; study with rhenium-188 suggested	[185]
- ^99m^Tc, ^177^Lu -tris(2-mercaptoethyl)-amine, isocyanobutyric acid succinimidyl ester, DOTA - x - various SP analogues	- preclinical in vitro - glioblastoma - x - lipophilic conjugates with specific tumor binding, high stability in buffer solutions, but lower stability in human serum	[186]
- ^64^Cu, ^67^Ga - NOTA - x - NK_1_R antagonist	- preclinical in vitro, in vivo - kidney - PET/CT - high in vivo stability, tumor uptake and good liver and renal clearance of [^64^Cu]Cu-NOTA-conjugate	[187]
- ^68^Ga - NODAGA - PEG_x_ - BBN(7-14), PACAP-27	- preclinical in vitro - x - improved stability of heterobivalent conjugates and comparable uptakes in tumor cells to those of monomers, further evaluation for in vivo PET/CT in progress	[188]
- ^68^Ga - NODAGA, DOTA - x - PACAP-27	- preclinical in vitro, in vivo - breast - PET/CT - low in vivo stability, but greater VPAC-affinity and tumor delineation only for NODAGA-conjugate	[189]
- ^64^Cu - N_2_S_2_ chelator - x - TP3805	- preclinical in vitro, in vivo - brain - microPET/CT - more specific brain tumor delineation than [^18^F]FDG, further investigation for clinical translation warranted	[190]

**Table 13:** Summary of radiolabeled small peptide inhibitors for PSMA- and FAP-positive tumor imaging over past 3 years.

Composition of Studied Compounds - Metal Radionuclide - BFCA - Linker - Peptide	Results and Findings - Phase of Trials - Cancer Type Studied - Imaging Technique Used - Benefits/Limitations/Conclusion	Reference
- ^68^Ga - THP - Ahx - EuK motif (PSMA)	- clinical, 118 patients - prostate - PET/CT - PET/CT impacts on management decisions in high-risk prostate cancer prior to radical therapy and biochemical recurrence	[214]
- ^99m^Tc - HYNIC - Gly-Ala-Asp-NaphthylAla - PSMA	- preclinical in vitro, in vivo - prostate - SPECT/CT - [^99m^Tc]Tc-HYNIC-conjugate with approximately similar pharmacokinetic and binding properties to [^68^Ga]Ga-PSMA-11, and great SPECT/CT visualization of tumor	[215]
- ^64^Cu - cyclam derivatives - naphtylAla, cyclohexane-carboxylic acid - PSMA	- preclinical in vitro, in vivo; first patient - prostate - PET - [^64^Cu]Cu-CA003 applied to first patient due to the best pharmacokinetic and imaging properties	[216]
- ^68^Ga - HBED-CC - Ahx - PSMA	- clinical - glioblastoma multiforme (GBM) - PET/CT - [^68^Ga]Ga-PSMA-11 as a highly promising agent for diagnosis of recurrent disease in patients with GBM due to low tumor-to-liver ratio and increased accumulation in recurrent lesions	[217]
- ^64^Cu, ^67^Cu - MeCoSar derivative - iodophenyl-1,2,3-triazolyl derivative - PSMA	- preclinical in vitro, in vivo - prostate - microPET/CT - Cu-labeled agents as promising alternatives to ^68^Ga-/^177^Lu-analogues in centers with limited access to these ligands	[77]
- ^68^Ga, ^89^Zr - DFO squaramide - p-aminomethylbenzoic acid - PSMA	- preclinical in vitro, in vivo - prostate - PET/CT - improved tumor uptake of bivalent inhibitors with 2 EuK motifs, ^89^Zr-complex as a promising alternative to ^68^Ga-analogue	[89]
- ^44^Sc - AAZTA derivatives - naphtylAla - PSMA	- preclinical in vitro, in vivo - prostate - PET/MRI - dynamic PET images showed high tumor uptake, rapid clearance from investigated tissue, very low accumulation at 150 min post-injection in the abdominal organs, lung, heart and brain, but higher in bladder	[218]
- ^68^Ga, ^177^Lu - DOTA - piperazine - FAPI-02,-04	- clinical, 23 patients together - fibrosarcoma, pancreatic, breast, lung, colon, thyroid, head and neck - microPET, PET/CT - [^68^Ga]Ga-FAPI-02 with TBR equal to or even better than [^18^F]FDG, PET/CT with ^68^Ga-probes can be performed without fasting and resting time	[219,220,221]
- ^68^Ga - DOTA - piperazine - FAPI-04	- clinical, 80 patients - 28 different tumor entities - PET/CT - the highest uptake in breast, esophagus, lung, pancreatic, head-neck, and colorectal cancer; FAPI limitations similar to those of FDG for renal and thyroid cancer	[222]
- ^68^Ga - DOTA - piperazine - FAPI-02/-04	- preclinical in vitro, in vivo; clinical, 18 patients - glioma - microPET, PET/CT - IDH-wildtype glioblastomas and grade III/IV, but not grade II IDH-mutant gliomas showed elevated tracer uptake	[223]
- ^64^Cu - DOTA - piperazine - FAPI-04	- preclinical in vitro, in vivo - pancreatic - microPET/CT - in vivo accumulation in tumor or normal organs significantly higher for [^64^Cu]Cu-FAPI-04 than [^68^Ga]Ga-FAPI-04, except in the heart	[224]
- ^68^Ga - DOTA - piperazine - FAPI-04	- clinical, 17 patients - hepatic - PET/CT - high sensitivity in poorly differentiated hepatic tumors	[225]
- ^68^Ga - DOTA - diazabicyclo[2.2.1]heptan containing linker - FAPI-46	- clinical, 6 patients - different tumor types - PET/CT - high TBR increasing over time and favorable dosimetry profile (highest effective doses were in bladder wall, ovaries, red marrow)	[226]
- ^68^Ga - DOTA - piperazine - FAPI-02/-04	- clinical, 13 patients - glioblastoma - PET/MRI - MRI- and FAP-specific gross tumor volumes were not congruent	[227]
- ^68^Ga - DOTA - piperazine - FAPI-04	- clinical, 68/75 patients - different tumor types - PET/CT - higher TBR of FAPI compared to FDG for brain metastases, FAPI identified more lesions for hepatic and peritoneal tumor manifestations, and had higher sensitivity in a detection of lymphonodal, osseous and visceral metastases	[228,229]
- ^99m^Tc - O*t*Bu-imidazol containing BFCA - piperazine - FAPI-19/-34	- preclinical in vitro, in vivo; clinical, 2 patients - pancreatic, ovarian - SPECT - [^99m^Tc]Tc-FAPI-34 accumulation in tumor lesions similar to [^68^Ga]Ga-FAPI-46	[230]

**Table 14:** Summary of radiolabeled small ligands for CA IX-positive tumor hypoxia imaging over past 3 years.

Composition of Studied Compounds - Metal Radionuclide - BFCA - Linker - Biomolecule	Results and Findings - Phase of Trials - Cancer Type Studied - Imaging Technique Used - Benefits/Limitations/Conclusion	Reference
- ^111^In - DOTA-ester - x - bis-ureidosulfonamide derivative	- preclinical in vitro, in vivo - breast, colorectal - SPECT/CT - rapid clearance from blood and muscle, and selective accumulation within CAIX expressing colon cancer cells	[240]
- ^99m^Tc - dipyridylamine, IDA - x - sulfonamide, sulfocoumarin	- preclinical in vitro, initial in vivo - colorectal - x - significant limitations in very low tumor uptake and much higher liver uptake	[241]
- ^68^Ga - CBT, NODA, pyridine, DOTA-NHS, NODAGA-NHS - Asp-Arg-Asp, PEG_2_ linker - acetazolamide	- synthesis, initial in vitro - x - useful CBT/1,2-aminothiol click reaction for CAIX ligands with in vitro stability developed	[113]
- ^99m^Tc - hydroxamamide (Ham), methyl-substituted-Ham (MHam) - x - sulfonamide, ureidosulfonamide	- synthesis, initial in vitro, in vivo - renal and colorectal - gamma counter - [^99m^Tc]Tc-MHam-bivalent conjugate with the highest tumor specificity useful for further studies	[242]
- ^111^In -DOTA-bis(tBu)ester - x - imidazothiadiazole sulfonamide	- preclinical in vitro, in vivo - breast and colorectal - SPECT/CT - favorable in vivo properties of [^111^In]In-DO3A-IS1 with selective binding and accumulation in CAIX-expressing colon cancer	[243]

## Data Availability

No new data were created or analyzed in this study. Data sharing is not applicable to this article.

## Abbreviations

AA	amino acid
AAZTA	6-amino-6-methylperhydro-1,4-diazepinetetraacetic acid
AHDA	amino-hexanedioic-1-acid
AHX	6-aminohexanoic acid
APCA	2-aminoethyl-piperazine-1-carboxylic acid
BBN	bombesin
BFCA	bifunctional chelating agent
hCA	human carbonic anhydrase
CBT	2-cyanobenzothiazole
CCK(R)	cholecystokinin (receptor)
c(RGDfK)	cyclo(-Arg-Gly-Asp-d-Phe-Lys)
CT	computed tomography
CuAAC	Cu(I)-catalyzed azide-alkyne cycloaddition
DATA	6-amino-1,4-diazepine-triacetate
DOTA	2,2′,2″,2‴-(1,4,7,10-tetraazacyclododecane-1,4,7,10-tetrayl)tetraacetic acid
DOTANOC	DOTA-Nal^3^-octreotide
DOTATATE	DOTA-Tyr^3^-octreotate
DOTATOC	DOTA-Tyr^3^-octreotide
DPA	5-(bis(pyridin-2-yl)methyl)amino)pentanoic acid
DTPA	diethylenetriamine pentaacetic acid
EDDA	ethylenediamine diacetic acid
EDTA	ethylenediamine tetraacetic acid
FAP	fibroblast activation protein
GE11	Tyr-His-Trp-Tyr-Gly-Tyr-Thr-Pro-Gln-Asn-Val-Ile
GEP NET	gastroenteropancreatic neuroendocrine tumors
GLP	glucagon-like peptide
GRPR	gastrin-releasing peptide receptor
HBED-CC	*N*,*N*′-*bis*-[2-hydroxy-5-(carboxyethyl)benzyl]ethylenediamine-*N*,*N*′-diacetic acid
HIF	hypoxia inducible factor
HPLC-DAD	high performance liquid chromatography-diode array detection
HYNIC	6-hydrazinopyridin-3-carboxylic acid
IDA	iminodiacetic acid
IEDDA	inverse electron-demand Diels-Alder reaction
JR11	p-Cl-Phe-cyclo(d-Cys-Aph(Hor)-d-Aph(cbm)-Lys-Thr-Cys)d-Tyr-NH_2_
MAG3	mercaptoacetylglycylglycylglycine
MC	melanocortin (receptor)
MG11	[3-MP^0^-d-Glu^1^,desGlu^2−6^]-Ala-Tyr-Gly-Trp-Met-Asp-Phe-NH_2_
MRI	magnetic resonance imaging
NeoBOMB1	d-Phe-Gln-Trp-Ala-Val-Gly-His-NH-CH[CH_2_-CH(CH_3_)_2_]_2_
NET	neuroendocrine tumors
NHS	N-hydroxysuccinimidyl-ester
NKR	neurokinin receptor
NODAGA	1,4,7-triazacyclononane-1-glutaric acid-4,7-diacetic acid
NODAPA	1,4,7-triazacyclononane-1,4-diacetic acid-7-p-phenylacetic acid
NODASA	1,4,7-triazacyclononane-1-succinic acid-4,7-diacetic acid
NOTA	2,2′,2″-(1,4,7-triazanonane-1,4,7-triyl)triacetic acid
NOTP	1,4,7-Triazacyclononane-1,4,7-tri(methylene phosphonic acid)
NP	nanoparticle
NSCLC	non-small cell lung cancer
NT(R)	neurotensin receptor
PA1	cyclo[HyPro-Phe-d-Trp-Lys-Tyr(Bzl)-Phe]
PAC-1	procaspase-activating compound receptor
PACAP	pituitary adenylate cyclase-activating peptide
PCTA	3,6,9,15-tetraazabicyclo[9.3.1]pentadeca-1(15),11,13-triene-3,6,9-triacetic acid
PEG	polyethylene glycol
PET	positron emission tomography
PC	prostate cancer
PSMA	prostate-specific membrane antigen
RGD	arginine-glycine-aspartic acid
RM26	1,4,7-triazacyclononane-N,N,N-triacetic acid-d-Phe-Gln-Trp-Ala-Val-Gly-His-Sta-Leu-NH_2_
RM2	DOTA-4-amino-1-carboxymethylpiperidine-d-Phe-Gln-Trp-Ala-Val-Gly-His-Sta-Leu-NH_2_
SERRS	surface-enhanced resonance Raman scattering
SPAAC	strain-promoted azide-alkyne cycloaddition
SPECT	single-photon emission computed tomography
SPPS	solid phase peptide synthesis
SST(R)	somatostatin (receptor)
TBR	tumor-to-background ratio
TETA	2,2′,2″,2‴-(1,4,8,11-tetraazacyclotetradecane-1,4,8,11-tetrayl)tetraacetic acid
THP	tris(hydroxypyridinone)
TMSAla	(l)-trimethylsilylalanine
TOC	Tyr^3^-octreotide
TPPTS	trisodium triphenylphosphine-3,3′,3″-trisulfonate
TRAP	1,4,7-triazacyclononane-1,4,7-tris(methylene(2-carboxyethylphosphinic acid))
UV	ultraviolet (detection)
VIP	vasoactive intestinal peptide
VPAC	receptor for vasoactive intestinal peptide
α-MSH	α-melanocyte stimulating hormone
